# Numerical simulation of roadway deformation and failure under different degrees of dynamic disturbance

**DOI:** 10.1038/s41598-022-24128-2

**Published:** 2022-11-21

**Authors:** Ce Jia, Sheng Li, Chaojun Fan, Hai Rong, Lei Yang, Ziang Pu

**Affiliations:** College of Mining, Liaoning Technical University, Fuxin, 123000 China

**Keywords:** Coal, Civil engineering

## Abstract

Deformation and failure of the roadway surrounding rock under dynamic disturbance were explored, which is essential for the control of the surrounding rock. The impact of dynamic disturbance on the deformation and failure of the roadway surrounding rock was studied from a single factor perspective using numerical simulation software. The disturbance intensity, frequency, and time were determined to affect the deformation and plastic zone of the surrounding rock. Firstly, a multi-factor integrated study was achieved using an orthogonal experimental design, and the impact of the three factors on the deformation and plastic zone of the surrounding rock were studied by applying mean value and extreme difference. The results show that the degree of influence of deformation of the roof is time > intensity > frequency in order. The impact of the plastic zone volume is intensity > frequency > time in order. Finally, a multivariate regression model was established using multiple regression analysis. The *P* = 0 < 0.05 for the regression model is obtained by variance analysis, and the equation regression is significant, which can effectively predict the deformation and failure of the surrounding rock under dynamic disturbance.

## Introduction

With the gradual depletion of the earth's external mineral resources, mining deep mineral resources have become the norm^1,2^. After coal enters deep mining, the mining environment becomes more complicated^3,4^. The surrounding rock is not only in a state of high stress but also encounters dynamic disturbances caused by blasting, fault slip, and mechanical vibration^5,6^. At this time, the dynamic disturbance may induce instability of the surrounding rock of the roadway or even rock burst^7,8^. Studying the influencing factors of surrounding rock deformation of the roadway under dynamic disturbance conditions is significant which is essential to control deep surrounding rock.

The deformation of the surrounding rock of the roadway is affected by the dynamic disturbance. At present, for rock dynamics testing^9,10^, domestic and foreign scholars^11–16^ mainly use drop hammer impact test devices^17^ and split Hopkinson pressure bar test devices^18^ to test the dynamic mechanical properties of coal and rock. For example, Baranowski^19^ and Michał Kucewicz^20^ respectively tested the mechanical properties of dolomite by using a drop hammer impact test device and SHBP device and verified by LS-DYNA numerical simulation software^21^. Tang^22^ used a split Hopkinson Pressure Bar (SHPB) experimental device to study coal’s dynamic mechanical response and energy dissipation behavior under impact load. Zhao^23^ used a self-made drop-hammer coal-rock impact loading test device to analyze the mechanism of the impact load on the evolution of the internal microstructure of the briquette. However, dynamic disturbance cannot be accurately measured in the field, and numerical simulation software mainly studies the impact of dynamic disturbance on surrounding rock. Liu^24^ studied the deformation and failure law of the surrounding rock of the deep super-large cross-section chamber group under different dynamic load intensity conditions using the finite difference software (Fast Lagrangian Analysis of Continua 3D, FLAC3D). Li^25^ used Particle Flow Code 2D (PFC2D) software to simulate the stability of high-stress roadways under dynamic loads and analyzed the instability and rupture of high-stress roadways induced by disturbance waves. Wang^26^ conducted a numerical simulation study on the cracking mechanism of roadway roof under cyclic impact load through the self-developed continuous-discontinuous method of Lagrangian yen and discrete element coupling. Xiao^27^ analyzed the evolution law of horizontal stress and elastic strain energy density of deep-buried roadway floor through FLAC3D software and concluded that the horizontal stress of roadway floor is the main inducing factor of floor impact.

The above study considered a single factor and the FLAC^3D^ software was unable to display the plastic zone volume values. However, dynamic disturbance is complex and affected by many factors. In this paper, the control variable method is used to verify the influence of the selected factors (intensity, frequency, time) on the deformation and failure of roadway surrounding rock. The volumes of shear_now,shear_past, tension_past are obtained by self-coding in FISH language. The orthogonal test was designed to achieve a multifactorial study. The sensitivity affecting the deformation and failure of the roadway surrounding was analyzed by mean value and extreme difference. Finally, a multiple linear regression model was established to effectively predict the deformation and failure of the surrounding rock under the dynamic disturbance effect.

## Numerical simulation of roadway deformation and failure under single factor

**Figure 1:**
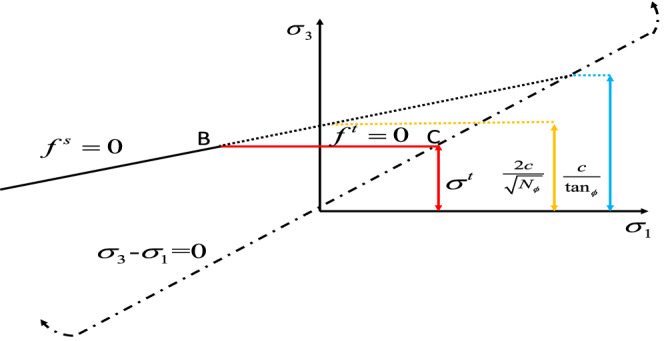
The failure criterion of the Moore-Coulomb model.

To explore the influencing factors of dynamic disturbance roadway failure and deformation, the FLAC3D dynamics module was used for numerical simulation. The rock layer’s thickness and the roadway’s size are based on a specific mine’s geological data. The 2D model is established by AUTOCAD software and saved in dxf format. The FLAC3D software provides the snap function for dxf format, which automatically captures the position of points. The 2D model was drawn using the Point-edge Tool, and the 3D model was extruded using the Extrusions function. According to the research of Kuhlemeyer and Lysmer^28^, a reasonable grid size was calculated, and a 3D model of 40 m × 20 m × 40 m was established. The deformation and failure of the roadway surrounding rock belong to the plane strain problem^29^. The A–B line was set in the y direction of the 3D model, and the 2D model was formed by slicing along the A–B line for research. Cai^30^ proposed that coal rock's elastic modulus, cohesion, and tensile strength can be estimated to be 0.1–0.25 times that of laboratory measurements. Poisson’s ratio can be calculated at 1.2–1.4 times that of laboratory measurements. The mechanical parameters of coal and rock were measured in the laboratory. Make assumptions about the model: (1) The influence of factors such as lithology and coal rock mass fracture is not considered. (2) The model is isotropic and conforms to the Mohr–Coulomb failure criterion^31^. This paper uses the Mohr–Coulomb constitutive model^32^ for the simulation. The rock mechanics parameters used in the numerical simulation are shown in Table 1. The constitutive model conforms to the Mohr–Coulomb failure criterion^33^, as shown in Fig. 1.

**Table 1:** Basic mechanical parameters.

Rock formation	Density (g·cm^−3^)	Bulk modulus (GPa)	Shear modulus (GPa)	Cohesion (MPa)	Internal friction angle (°)	Tensile intensity (MPa)
Basic roof	2.6	1.84	0.80	2.3	28	2.0
Immediate roof	2.5	1.79	0.97	1.9	30	1.6
Coal	1.4	1.66	0.85	2.1	32	1.28
Immediate floor	2.5	1.79	0.97	1.9	30	1.6
Basic floor	2.6	1.84	0.80	2.3	28	2.0

When $f^{s} = 0$, the material occurs shear failure. When $f^{t} = 0$, the material occurs tensile failure. The Mohr–Coulomb destruction criterion expression^34^:

1 $$ \left\{ \begin{gathered} f^{s} = \sigma_{1} - \sigma_{3} N_{\phi } + 2c\sqrt {N_{\phi } } \hfill \\ f^{t} = \sigma_{3} - \sigma^{t} \hfill \\ \end{gathered} \right. $$

where $\sigma_{1}$ is the maximum principal stress; $\sigma_{3}$ is the minimum principal stress; $c$ is material cohesion; $\phi$ is the friction angle; $\sigma^{t} \left( {\sigma_{\max }^{{\text{t}}} { = }\frac{c}{\tan \phi }} \right)$ is tensile intensity; $N_{\phi } { = }\frac{{1{\text{ + sin(}}\phi {)}}}{{1 - {\text{sin(}}\phi {)}}}$.

Firstly, the model is statically balanced, the left and right boundaries of the model are displacement constraints, and the bottom boundary is constrained. The upper boundary is free and bears the uniform load applied to this boundary by the overburden^35^.

According to the literature^36–38^, the shock wave received by the roadway is simplified into a half-sine wave, which is written in the Fish language and embedded in the dynamic calculation process. The normal stress wave expression used in this paper is as follows:

2 $$ F = \left\{ {\begin{array}{*{20}l} {1/2\sigma (1 - \cos (2\pi ft))} \hfill & {(t < 1/f)} \hfill \\ 0 \hfill & {(t > 1/f)} \hfill \\ \end{array} } \right. $$

where $\sigma$ is the disturbance intensitie, MPa; $f$ is the disturbance frequency, Hz; $t$ is the disturbance time, s.

Since the intensity, frequency, and time of dynamic disturbance are difficult to measure under the existing technical conditions, the intensity, frequency, and time values in this paper cover the current literature^39–41^, as shown in Table 2.

**Table 2:** Numerical simulation test factors and value ranges.

Factor	Ranges
Disturbance intensity	6 MPa, 9 MPa, 12 MPa, 15 MPa
Disturbance frequency	5 Hz, 10 Hz, 15 Hz, 20 Hz
Disturbance time	0.2 s, 0.4 s, 0.6 s, 0.8 s

**Figure 2:**
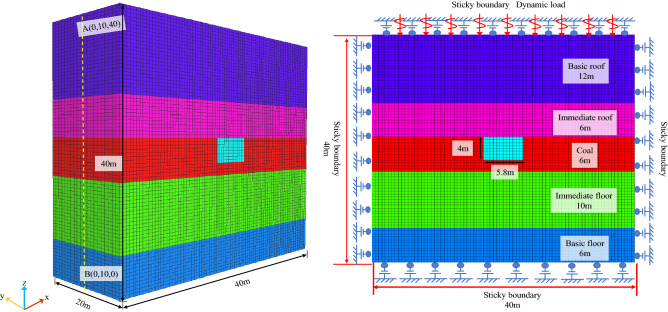
Geometric model and dynamic boundary conditions.

After static equilibrium, the model is solved by roadway excavation until equilibrium, and then the dynamic calculation is performed. According to the literature^42^, the Ruili damping is selected, the minimum damping ratio is 0.3%, and the center frequency is 5.2 Hz. In order to reduce stress wave reflection, sticky boundaries are used around the model to absorb normal incident waves, as shown in Fig. 2.

### Numerical simulation of roadway deformation and failure under different disturbance intensity

**Figure 3:**
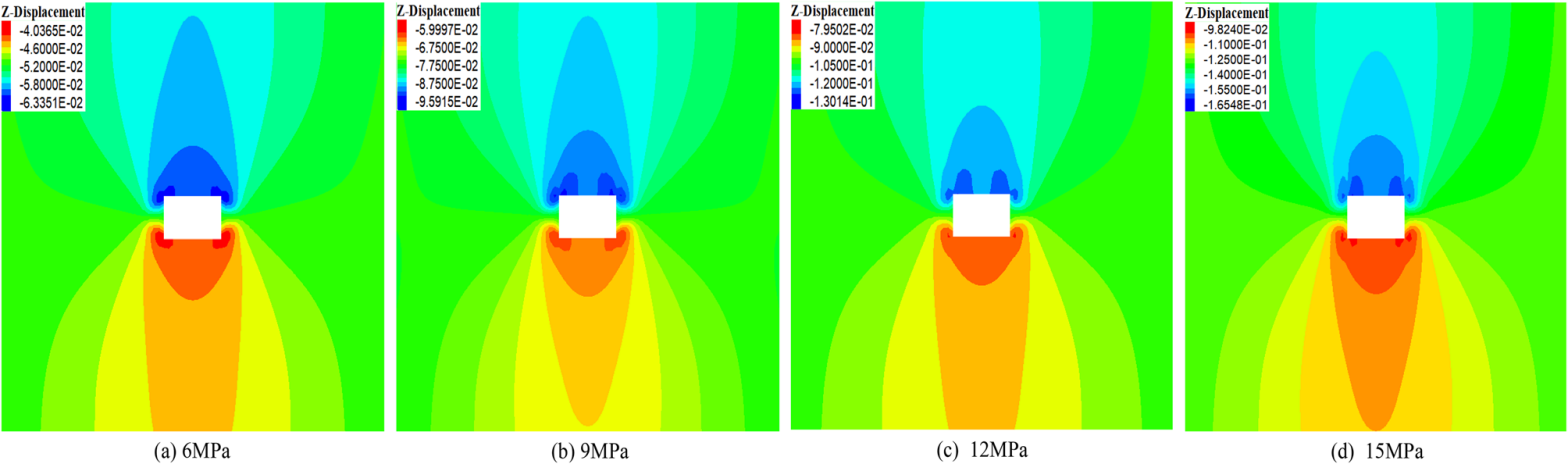
Deformation of surrounding rock under different disturbance intensities.

The control variable method is used to fix the disturbance time and frequency, and the disturbance intensity is changed to study the deformation and failure of the surrounding rock. Figure 3 illustrates the deformation of surrounding rock under different disturbance intensities. In Fig. 3a, when the disturbance intensity is 6 MPa, the maximum displacements of the roof and the floor are 63 mm and 40 mm. In Fig. 3b, when the disturbance intensity increases from 6 to 9 MPa, the maximum displacement of the roof increases from 63 to 95 mm, with an increase of 50.7%. In Fig. 3c, when the disturbance intensity increases from 9 to 12 MPa, the maximum displacement increases from 95 to 130 mm, with an increase of 36.8%. In Fig. 3d, when the disturbance intensity increases from 12 to 15 MPa, the maximum displacement of the roof increases from 130 to 165 mm, with an increase of 27%. In summary, with the increase in disturbance intensity, the deformation of the surrounding rock gradually increases, and the effect of the increase shows a trend from large to small.

**Figure 4:**
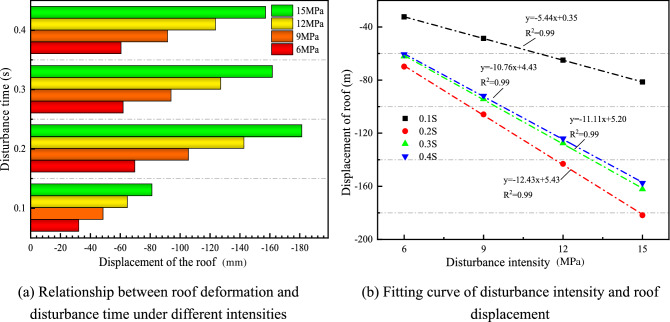
Variation law of roof displacement under different disturbance intensities.

Figure 4 illustrates the displacement of the roof under different disturbance intensities. In Fig. 4a, when the disturbance intensity is 15 MPa, the disturbance time increases from 0.1 to 0.4 s, and the displacement of the roof shows a trend of increasing–decreasing. When the disturbance intensity is 12 MPa, 9 MPa, and 6 MPa, all show the same direction, which is consistent with the conclusion of the literature^43^. The disturbance intensity is linearly fitted with the roof displacement, as shown in Fig. 4b. The R^2^ is close to 1, and the fitting effect is better.

**Figure 5:**
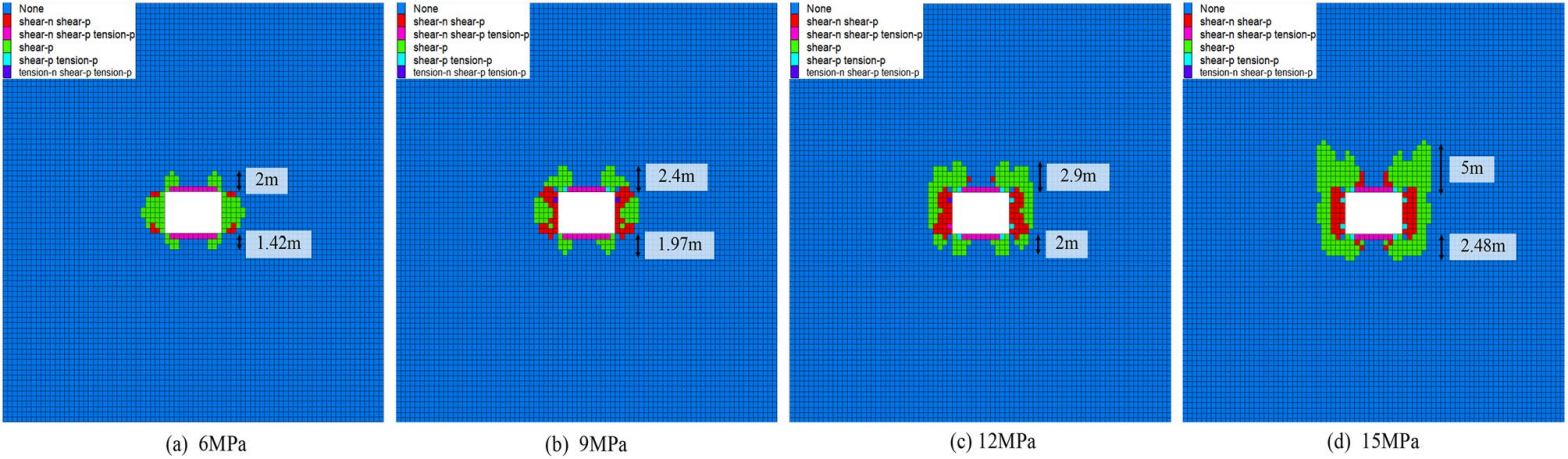
Failure of surrounding rock under different disturbance intensities.

Figure 5 illustrates the failure of the surrounding rock under different disturbance intensities. In Fig. 5a, when the disturbance intensity is 6 MPa, the failure range of the roof reaches 2 m, and the floor reaches 1.42 m. The failure of the roof is more severe in the dynamic disturbance. In Fig. 5(b), when the disturbance intensity is 9 MPa, the failure range of the roof increases to 2.4 m, with an increase of 20%. The failure range of the floor increased to 1.97 m, with an increase of 28%. In Fig. 5c, when the disturbance intensity increases from 9 to 12 MPa, the failure range of the roof increases from 2.4 to 2.9 m, with an increase of 20.8%. The failure range of the floor increased from 1.97 to 2 m, with an increase of 1.52%. In Fig. 5d, when the disturbance intensity increases from 12 to 15 MPa, the failure range of the roof increases from 2.9 m to 5 m, with an increase of 72.4%. The failure range of the floor increased from 2 to 2.48 m, with an increase of 24%. In summary, with the increase in dynamic disturbance intensity, the failure of the surrounding rock was transferred to the deeper part.

**Figure 6:**
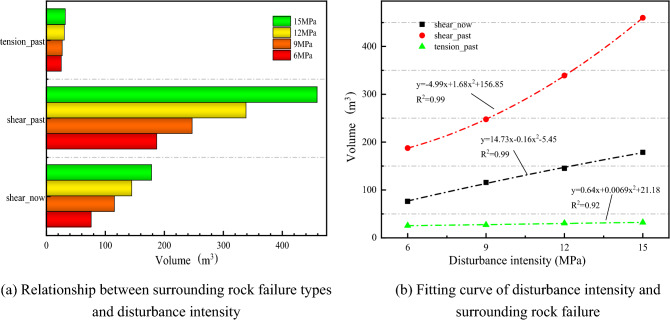
Variation law of surrounding rock failure types under different disturbance intensities.

Figure 6 illustrates the law of failure type of surrounding rock under different disturbance intensities. The Fish language was used to extract the volume of the three types of failure in the surrounding rock. In Fig. 6a, the three kinds of failure show an increasing trend with the increase in disturbance intensity. The dynamic disturbance of the surrounding rock changes the stress distribution, causing the stress to reach the Mohr intensity envelope, resulting in failure. The disturbance intensities were fitted with quadratic term interpolation polynomials with the three failure types, as shown in Fig. 6b. The R^2^ is close to 1, and the fitting effect is good.

### Numerical simulation of roadway deformation and failure under different disturbance frequencies

**Figure 7:**
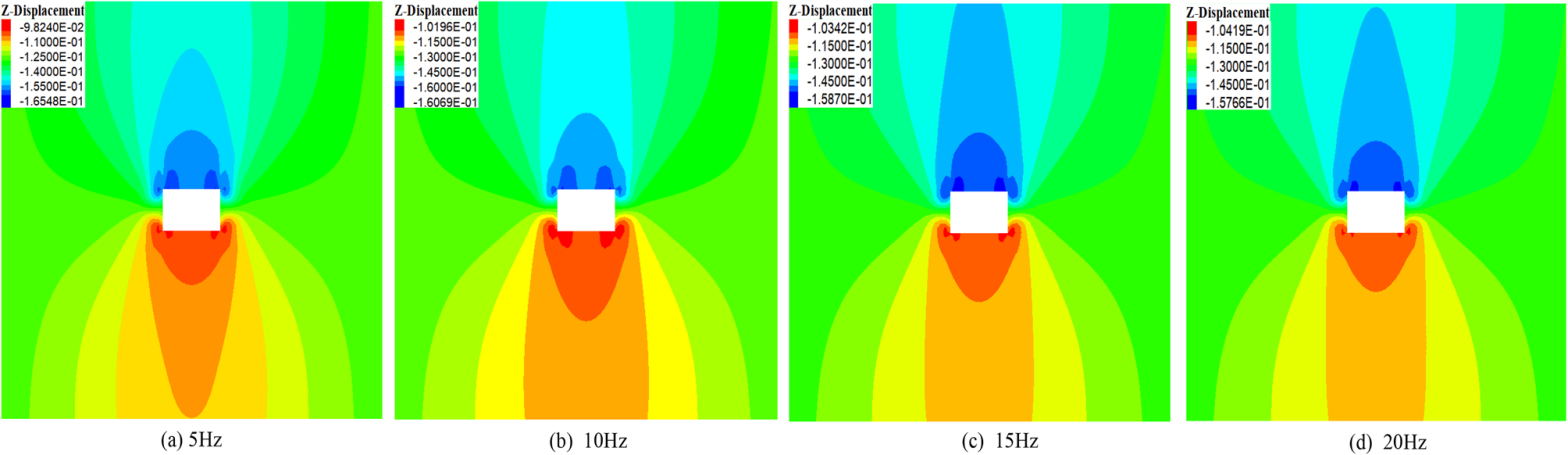
Deformation of surrounding rock under different disturbance frequencies.

Figure 7 illustrates the deformation of surrounding rock under different disturbance frequencies. In Fig. 7a, when the interference frequency is 5 Hz, the maximum displacement of the roof is 165 mm, and the maximum displacement of the floor is 98 mm. In Fig. 7b, when the disturbance frequency increases from 5 to 10 Hz, the maximum displacement of the roof decreases from 165 to 160 mm, with a reduction of 3.03%. The displacement of the floor increases from 98 to 101 mm. In Fig. 7c, when the disturbance frequency increases from 10 to 15 Hz, the maximum displacement of the roof decreases from 160 to 158 mm, with a reduction of 1.25%. The displacement of the floor increases from 101 to 103 mm. In Fig. 7d, when the disturbance frequency increases from 15 to 20 Hz, the maximum displacement of the roof decreases from 158 to 157 mm. The displacement of the floor increased from 103 to 104 mm. In summary, with an increase in disturbance frequency, the displacement of the roof decreases, and the displacement of the floor increases, but the change of both is insignificant.

**Figure 8:**
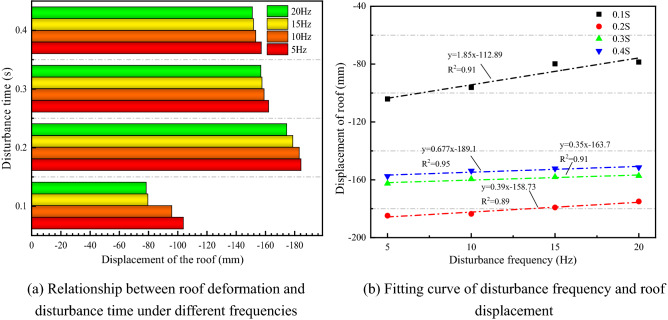
Variation rule of roof displacement under different disturbance frequencies.

Figure 8 illustrates the variation law of the roof displacement under different disturbance frequencies. In Fig. 8a, when the disturbance intensity and time are constant, the displacement of the roof decreases as the disturbance frequency increases. In summary, the lower the disturbance frequency, the greater the effect on the deformation of the surrounding rock, which is verified by the literature results^44^. When the disturbance frequency is specific, with the increase of time, the displacement of the roof shows an increasing–decreasing trend. The disturbance frequency is fitted linearly with the roof displacement, as shown in Fig. 8b. The R^2^ is close to 1, and the fitting effect is excellent.

**Figure 9:**
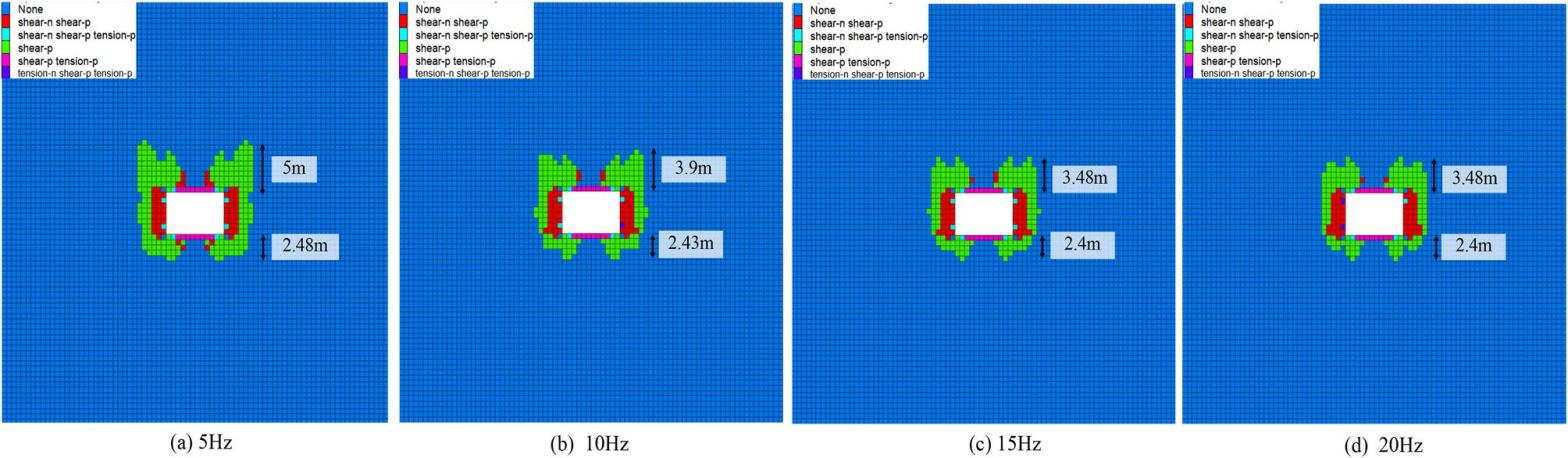
Failure of surrounding rock under different disturbance frequencies.

Figure 9 shows the failure of the surrounding rock with different disturbance frequencies. In Fig. 9a, when the disturbance frequency is 5 Hz, the failure range of the roof reaches 2 m, and the floor reaches 2.48 m. In Fig. 9b, when the disturbance frequency increases to 10 Hz, the failure range of the roof decreases to 3.9 m, with a reduction of 22%. The failure range of the floor is reduced to 2.43 m, with a decrease of 2.01%. The disturbance frequency greatly influences the failure of the roof. In Fig. 9c, when the disturbance frequency is increased from 10 to 15 Hz, the failure range of the roof is reduced from 3.9 to 3.48 m, with a reduction of 11%. In Fig. 9d, when the disturbance frequency is increased from 15 to 20 Hz, the failure range of the roof is the same as in Fig. 9c. In summary, when the disturbance intensity and time are specific, the disturbance frequency increases, and the failure range of the surrounding rock decreases.

**Figure 10:**
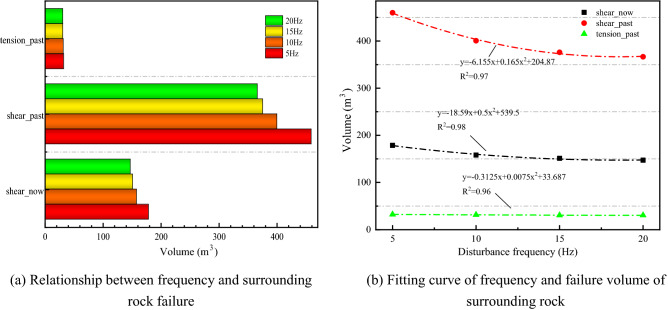
The law of surrounding rock failure types under different disturbance frequencies.

Figure 10 illustrates the law of failure type of surrounding rock under different disturbance frequencies. In Fig. 10a, the volume of the three failure types decreases when the disturbance frequency increases. The smaller the frequency of disturbance, the more serious the failure to the surrounding rock. The surrounding rock failure is mainly shear. Compared with Shear_past and tension_past, Shear_past has the most significant volume change. The quadratic polynomial fitting Fig. 10b. The R^2^ is close to 1, and the fitting effect is good.

### Numerical simulation of roadway deformation and failure under different disturbance time

**Figure 11:**
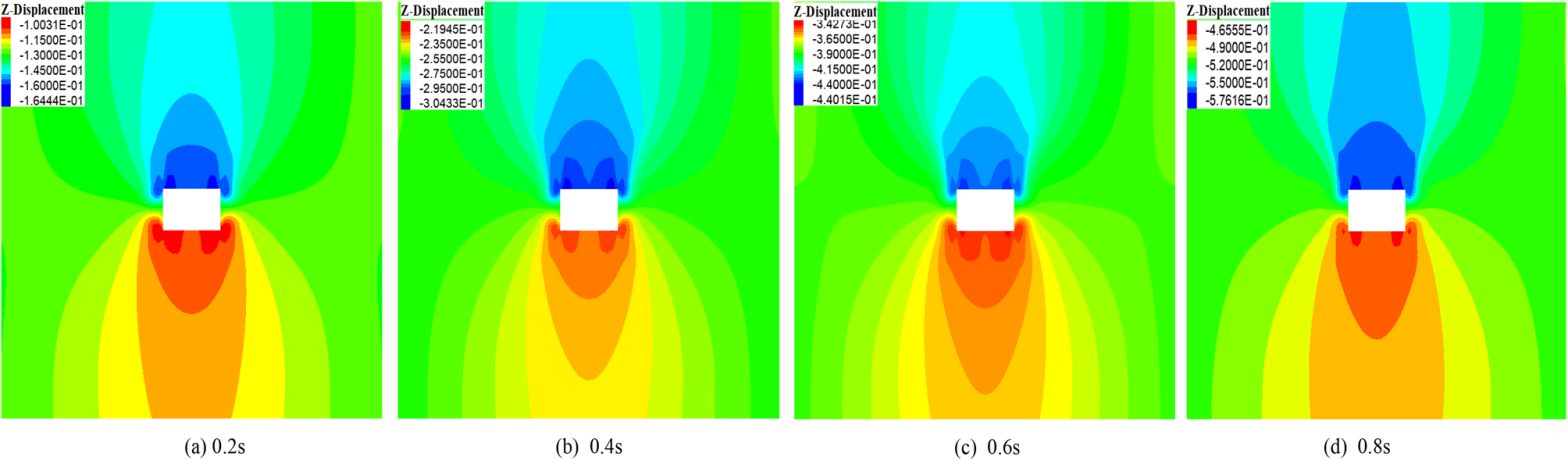
Deformation of surrounding rock under different disturbance times.

Figure 11 shows the deformation evolution law of the surrounding rock under different disturbance times. In Fig. 11a, when the disturbance time is 0.2 s, the maximum displacements of the roof and the floor are 164 mm and 100 mm. In Fig. 11b, when the disturbance time increases from 0.2 to 0.4 s, the maximum displacement of the roof increases from 161 to 304 mm, with an increase of 87%. The floor displacement increased from 100 to 219 mm, with an increase of 119%. In Fig. 11c, when the disturbance time increases from 0.4 to 0.6 s, the maximum displacement of the roof increases from 304 to 440 mm, with an increase of 45%. The displacement of the floor increased from 219 to 342 mm, with an increase of 56%. In Fig. 11d, when the disturbance time increases from 0.6 to 0.8 s, the maximum displacement of the roof increases from 440 to 575 mm, with an increase of 31%. The displacement of the floor increased from 342 to 465 mm, with an increase of 36%. In summary, with the increase in disturbance intensity, the deformation of the surrounding rock increases, and the increase of deformation displacement of the floor is more significant compared with the increase of displacement of the roof.

**Figure 12:**
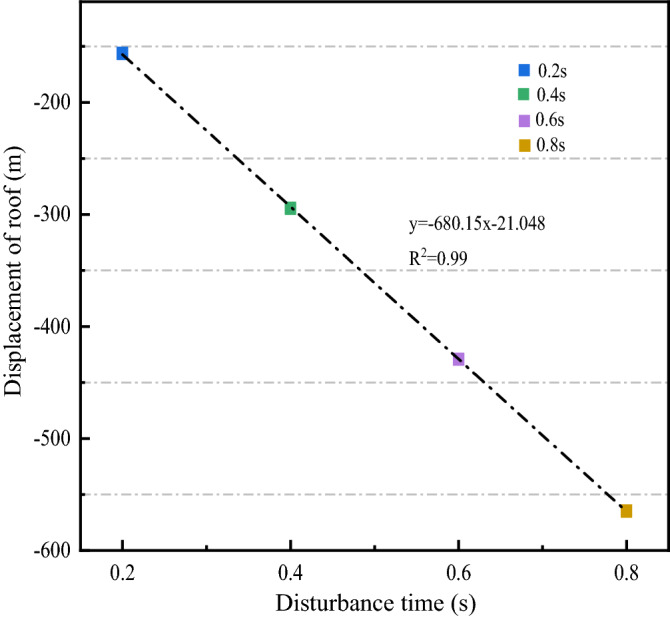
Fitting curve of different disturbance time and roof displacement.

Figure 12 illustrates the fitted curves for different disturbance times and the deformation of the roof. In Fig. 12, when the disturbance intensity and frequency are specific, the displacement of the roof increases linearly with the increase of the disturbance time, and the fitting equation is shown in Fig. 12.

**Figure 13:**
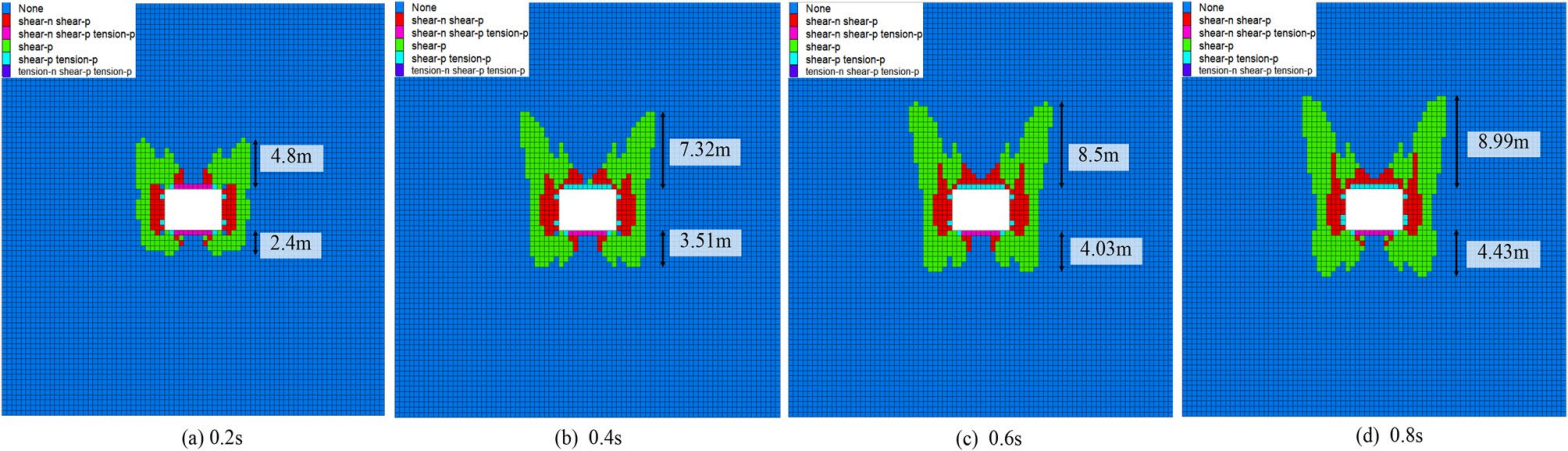
Failure of surrounding rock under different disturbance times.

Figure 13 shows the failure evolution law of the surrounding rock under different disturbance times. In Fig. 13a, when the disturbance time is 0.2 s, the failure range of the roof is 4.8 m, and the floor is 2.4 m. In Fig. 13b, when the disturbance time is increased from 0.2 to 0.4 s, the failure range of the roof increases from 4.8 to 7.32 m, with an increase of 52.5%. The failure range of the floor increased from 2.4 to 3.51 m, with an increase of 46.2%. In Fig. 13c, when the disturbance time is increased from 0.4 to 0.6 s, the failure range of the roof increases from 7.32 to 8.5 m, with an increase of 16.12%. The failure range of the floor increased from 3.51 to 4.03 m, with an increase of 15%. In Fig. 13d, when the disturbance time is increased from 0.6 to 0.8 s, the failure range of the roof increases from 8.5 to 8.99 m, with an increase of 6%. The failure range of the floor increased from 4.03 to 4.43 m, an increase of 10%. In summary, with the increase in disturbance time, the failure range of the surrounding rock increases. When the disturbance time increases from 0.2 to 0.4 s, the failure range of the surrounding rock increases the most. When the disturbance time increased from 0.2 to 0.4 s, the increasing effect of the whole process showed an increasing–decreasing trend.

**Figure 14:**
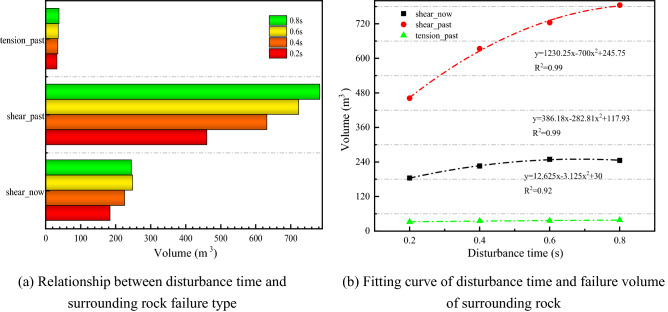
The law of surrounding rock failure types under different disturbance time.

Figure 14 illustrates the variation pattern of failure type of surrounding rock under different disturbance times. In Fig. 14a, the three categories of failure volumes show an increasing trend with the increased disturbance time. When the disturbance intensity and frequency are specific, the longer the continuous disturbance time, the more failure to the surrounding rock increases. The quadratic polynomial fit of the disturbance time to the failure volume of the surrounding rock is shown in Fig. 14b. The R^2^ is close to 1, and the fit is good.

## Numerical simulation of roadway deformation and failure under multi-factor conditions

The orthogonal design experiment method is a mathematical-statistical method suitable for multi-factor experiments^45^. In order to explore the failure and deformation of the roadway under the dynamic disturbance condition under the multi-factor condition, the orthogonal experiment method is adopted. Three main factors are selected to satisfy the factors and level uniformity: Disturbance intensity, frequency, time, and set four levels, respectively, as shown in Table 3. An L16 (4^3^) orthogonal experiment table was designed by Minitab software, as shown in Table 4.

**Table 3:** Factors and levels.

Factor	Intensity (A/MPa)	Frequency (f/Hz)	Time (T/s)
Level			
1	6	5	0.2
2	9	10	0.4
3	12	15	0.6
4	15	20	0.8

**Table 4:** Orthogonal experiment result.

Experiment number	Intensity (A/MPa)	Frequency (F/Hz)	Time (T/s)
1	1	1	1
2	1	2	2
3	1	3	3
4	1	4	4
5	2	1	2
6	2	2	1
7	2	3	4
8	2	4	3
9	3	1	3
10	3	2	4
11	3	3	1
12	3	4	2
13	4	1	4
14	4	2	3
15	4	3	2
16	4	4	1

**Figure 15:**
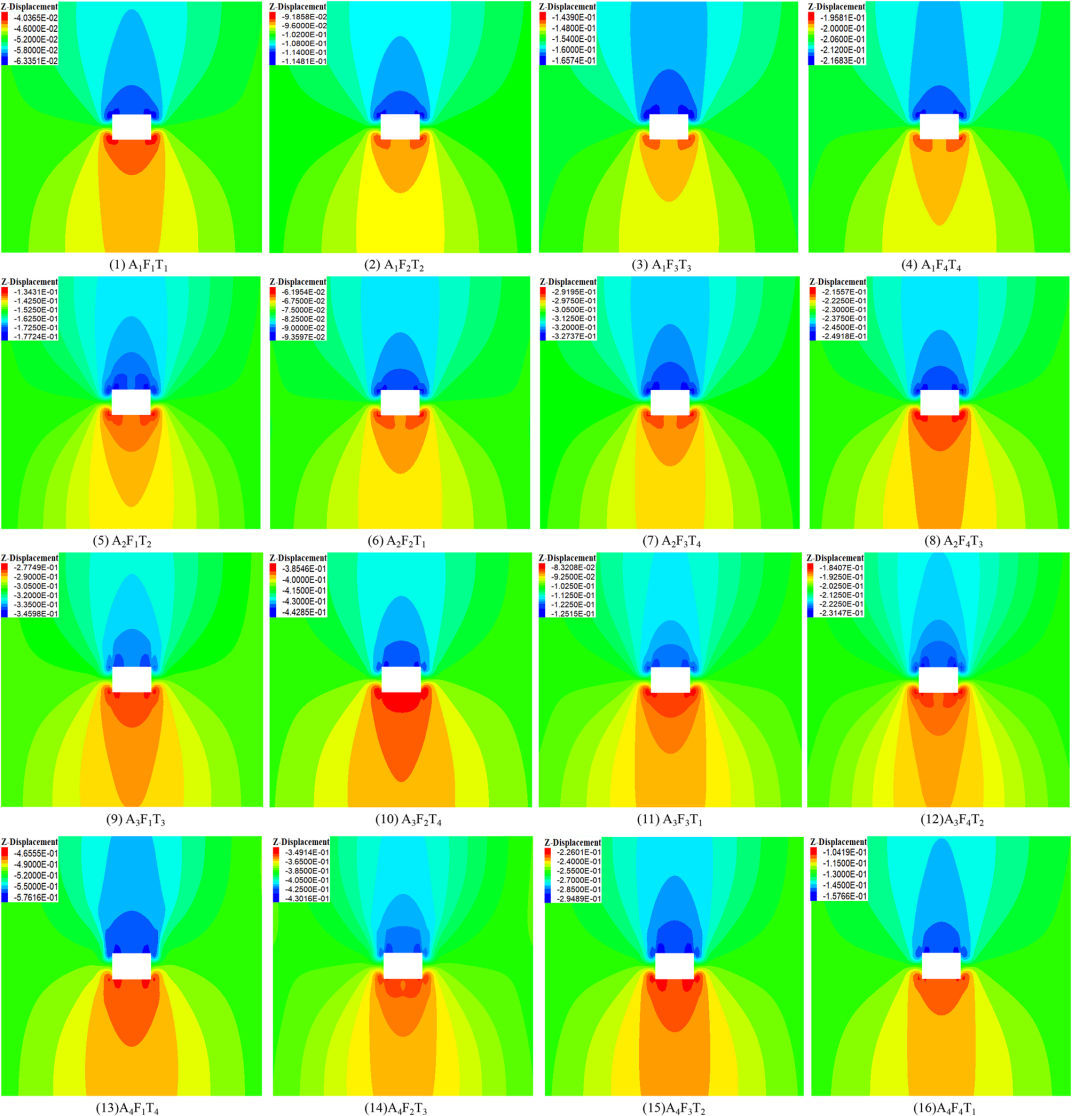
Deformation of surrounding rock under different factors.

The deformation and failure laws of the surrounding rock were obtained by orthogonal experiments. Figure 15 illustrates the deformation evolution law of the surrounding rock through 16 orthogonal experiments. From experiments 1 to 4, when the disturbance intensity is constant and the disturbance frequency and time increase, the maximum displacement of the roof increases. From experiments 5 to 8, when the disturbance intensity increases from 6 to 9 MPa, the disturbance frequency gradually increases, the disturbance time shows a decreasing–increasing–decreasing trend, and the maximum displacement of the roof also shows a decreasing–increasing–decreasing trend. The roof deformation trend from experiments 9 to 12 is the same as that of experiments 5 to 8. From experiments 13 to 16, the roof deformation gradually decreases. In summary, the disturbance time dramatically impacts the roof deformation.

**Figure 16:**
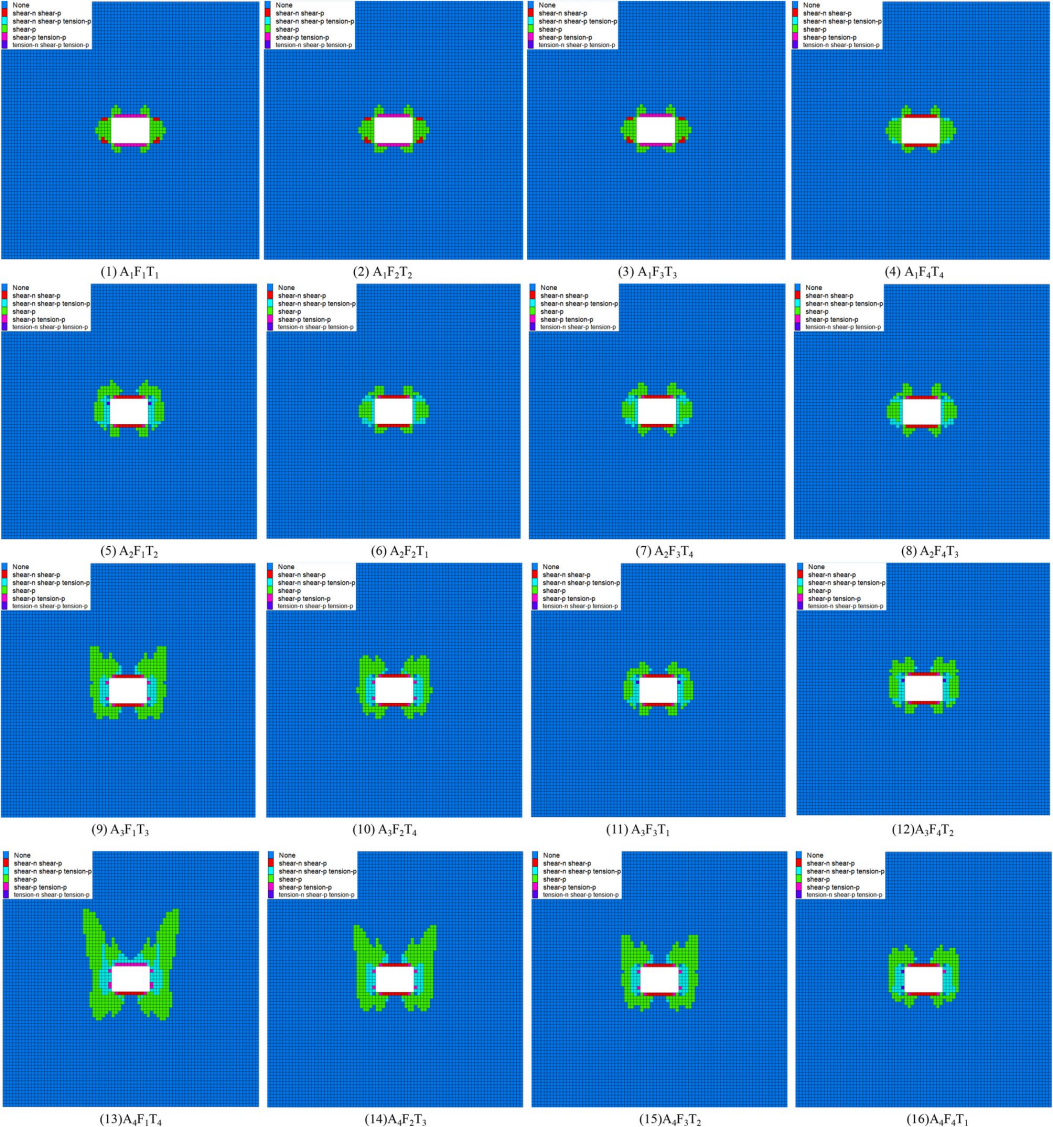
Failure of surrounding rock under the different factors.

Figure 16 illustrates the failure evolution law of the surrounding rock through 16 orthogonal experiments. In experiments 1 to 4, when the disturbance intensity is constant and the disturbance frequency and time increase, the plastic zone volume value increases, but the change is slight. From experiments 5 to 8, when the disturbance intensity increased from 6 to 9 MPa, the disturbance frequency gradually increased, the disturbance time showed a decreasing-increasing trend, and the plastic zone volume also showed a decreased-increasing trend, but the change was not significant. In experiments 9 to 12, the disturbance intensity increases by 12 MPa, the disturbance frequency gradually increases, the disturbance time shows an increasing–decreasing–increasing trend, and the plastic zone volume also shows an increasing–decreasing–increasing trend, and the variation increases. In experiments 12 to 16, the disturbance intensity was 15 MPa, the disturbance frequency gradually increased, the disturbance time gradually decreased, and the volume of the plastic zone gradually decreased and changed the most. In summary, the disturbance intensity dramatically impacts the volume of the plastic zone.

**Figure 17:**
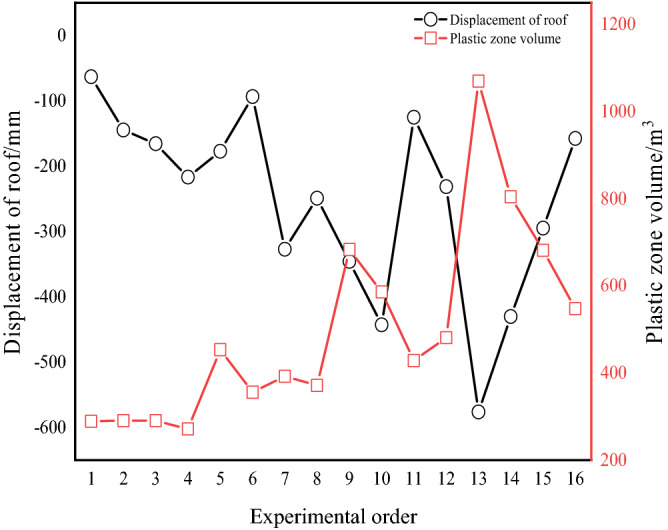
The maximum displacement of the roof and the volume of the plastic zone.

The maximum displacement of the surrounding rock roof and the volume value of the plastic zone in 16 orthogonal experiments were recorded using the self-developed Fish language, as shown in Fig. 17.

### Sensitivity analysis of roadway surrounding rock deformation and failure factors under dynamic disturbance

According to the maximum displacement of the roof and the Plastic zone volume obtained from the above orthogonal experiment, the mean value of each factor is calculated according to formulas (3) and (4) ^46^.

3 $$ K_{ij} = \sum\limits_{d}^{c = 1} {Y_{ijc} } $$

where *K*_*ij*_ is the statistical parameter of the result of factor *i* at the *j* level; *Y*_*ijc*_ (c = 1, 2, 3, …d) is the experimental result obtained in the experiment of group *c*.

4 $$ k_{i} = \max \left\{ {\overline{K}_{i1} ,\overline{K}_{i2} ,\overline{K}_{i3} ,\overline{K}_{i4} } \right\} - \min \left\{ {\overline{K}_{i1} ,\overline{K}_{i2} ,\overline{K}_{i3} ,\overline{K}_{i4} } \right\} $$

where *K*_*i*_ (*i* = A, B, C, …) is the range of the *i* factor; $\overline{K}_{{i{\text{j}}}}$ is the mean of the *j* level of factor *i*.

**Figure 18:**
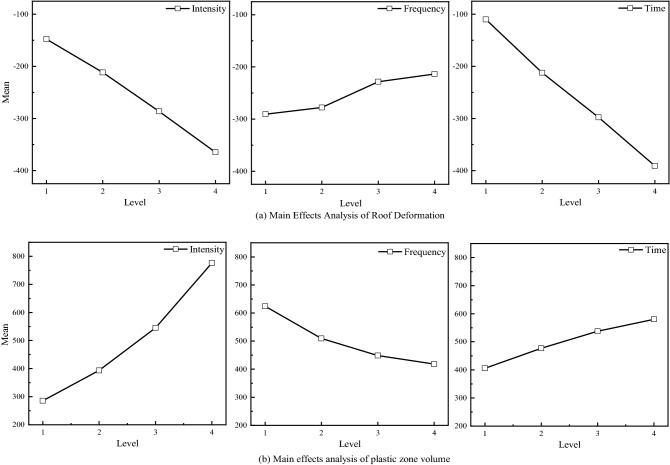
Main effect diagram of deformation and failure of surrounding rock.

Figure 18 shows the main effects of deformation and failure of the roadway surrounding rock. In Fig. 18a, with the increase of disturbance intensity, the deformation of the roof increases, and the two show a positive correlation. With the increase of disturbance frequency, the deformation of the roof decreases, and the two shows negatively correlated. With the increase of disturbance time, the deformation of the roof increases, and the two show a positive correlation. In Fig. 18b, with the increase of disturbance intensity, the plastic zone volume increases, and the two show a positive correlation. With the increase of disturbance frequency, the volume of plastic zone decreases, and the two show a negatively correlated. With the increase in disturbance time, the volume of the plastic zone decreases, and the two show a negatively correlated.

**Figure 19:**
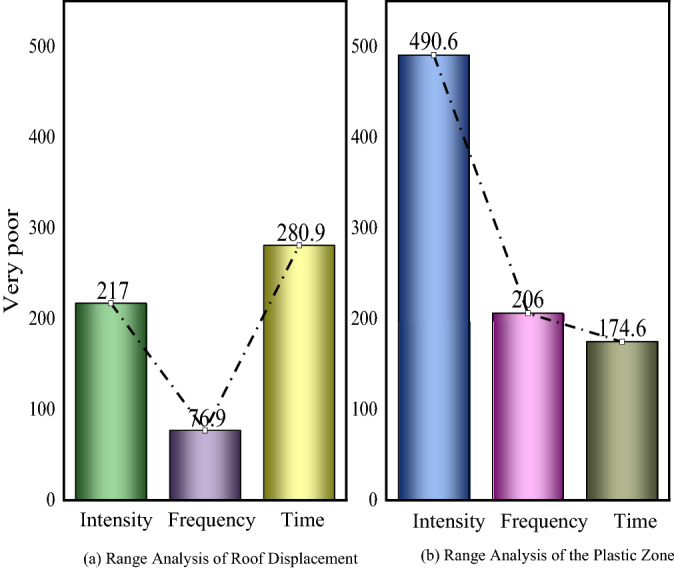
Factor sensitivity to deformation and failure of surrounding rock Supplementary Information.

Figure 19 shows the sensitivity of surrounding rock deformation and failure to factors. In Fig. 19a, the range of each factor is obtained according to formula (2), and the degree of influence on the top plate deformation is time > intensity > frequency in order. In Fig. 19b, the degree of influence on the volume value of the plastic zone is intensity > frequency > time in order.

## Regression model of surrounding rock failure and deformation under multi-factor dynamic disturbance

According to the orthogonal experiment results, a multiple linear regression mathematical model is established. Assuming that the influencing factors are *x*_1_, *x*_2_, *x*_3_, *x*_4_, … *x*_k_, and the corresponding experimental results are *y*_1_, *y*_2_, *y*_3_, *y*_4_, … *y*_k_, then the multiple linear regression model^47^:

5 $$ y = \beta_{0} + \beta_{1} x_{1} + \beta_{2} x_{2} + ... + \beta_{k} x_{k} + \varepsilon $$

The corresponding multiple linear regression equation is:

6 $$ \hat{y} = \hat{\beta }_{0} + \hat{\beta }_{1} x_{1} + \hat{\beta }_{2} x_{2} + ... + \hat{\beta }_{k} x_{k} $$

where *y* is the displacement and deformation of the surrounding rock, mm; $\beta_{0}$ is the regression constant; $\beta_{1}$, … $\beta_{k}$ is the regression model coefficient;$\hat{\beta }_{0}$, $\hat{\beta }_{1}$, … $\hat{\beta }_{2}$ $\hat{\beta }_{k}$ is the coefficient of the corresponding regression equation;$\varepsilon$ is the random error, mm.

Whether the regression model can truly reflect the relationship between *x*_*k*_ and *y*_*k*_ requires statistical testing. The multivariate regression model was tested for significance according to the statistic F value, and the calculated F value was compared with the lower critical value *F*_*α*_(k, n − k − 1) of the significant level *α* obtained by looking up the table^48^.

7 $$ F = \frac{{Q_{{\text{r}}} /k}}{{Q_{e} /(n - k - 1)}} $$

where *Qr* is the regression sum of squares,mm^2^; *Qe* is the error sum of squares,mm^2^.

### Multiple linear regression model and significance test of roof deformation

**Figure 20:**
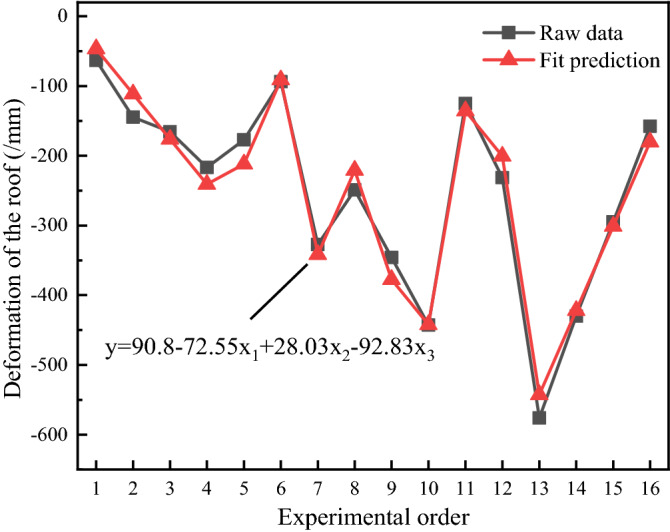
Comparison between the roof regression model and the original experimental data.

A multiple linear regression model of disturbance intensity, frequency, time and roof deformation was obtained using Minitab software. The data obtained from the orthogonal experiments (Table 4, Fig. 17) were imported into Minitab software for multiple linear regression analysis.The multiple linear regression model is y = 90.8 − 72.55x_1_ + 28.03x_2_ − 92.83x_3_, and the comparison between the roof regression model and the original experimental data is shown in Fig. 20.

Based on the above principles, the regression model for the roof deformation was tested for significance, as shown in Table 5. The overall significance of the regression equation is *P* = 0 < 0.05, indicating that at the significant level α = 0.05, the regression of the multiple linear regression model is significant. Table 5 illustrates that the *p* values of all three factors are less than 0.05. All three factors significantly affect the roof deformation of the roadway surrounding rock.

**Table 5:** Analysis of variance of the roof deformation regression model.

Source	Degrees of freedom	Adj SS	Adj MS	F	P
Regress	3	293,327	97,776	144.80	0.000
Intensity	1	105,277	105,277	155.91	0.000
Frequency	1	15,711	15,711	23.27	0.000
Time	1	172,339	172,339	255.23	0.000
Error	12	8103	675		
Total	15	301,430			

Adj SS is the adjusted sum of squares; Adj MS is the squared sum of deviations; F is the test value; *P* is the significant difference value.

**Figure 21:**
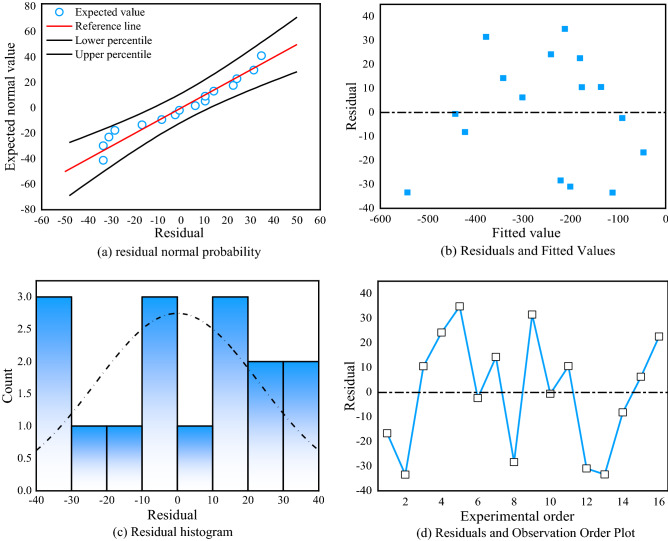
Residual plots of the roof regression model.

Figure 21 shows the residuals of the roof regression model. Figure 21a shows the residual normal probability plot, which conforms to a normal distribution and shows no outliers. Figure 21b shows a graph of residuals and observation order. The residuals fluctuate randomly, indicating that the residuals are independent of each other. Figure 21c shows the histogram of the residuals, which all conform to a normal distribution with no outliers. Figure 21d shows a graph of residuals and observation order, with random fluctuations in the residual values, indicating that the residuals are independent of each other. In summary, the regression of the roof deformation multiple regression model was statistically tested to be significant.

### Multiple linear regression model and significance test of plastic zone volume

**Figure 22:**
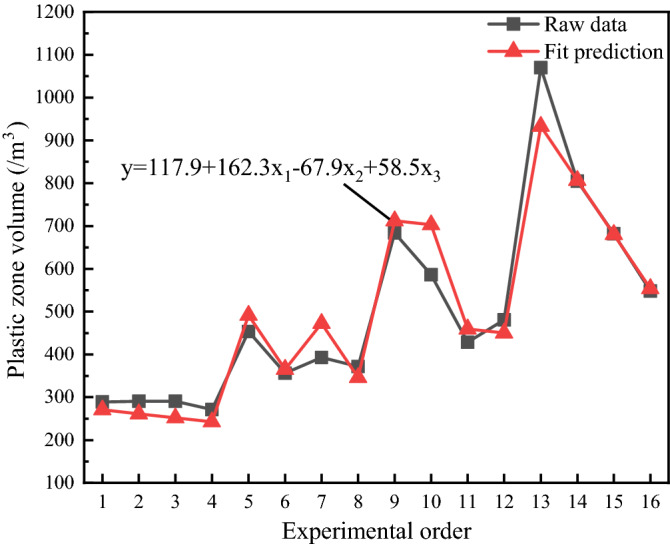
Comparison between the plastic zone volume regression model and the original experimental data.

A multiple linear regression model of disturbance intensity, frequency, time and plastic zone volume was obtained using Minitab software. The multiple linear regression model is y = 117.9 + 162.3x_1_ − 67.9x_2_ + 58.5x_3_, and the comparison between the plastic zone volume regression model and the original experimental data is shown in Fig. 22.

Based on the above principles, the regression model for the plastic zone volume was tested for significance, as shown in Table 6. The overall significance of the regression equation is *P* = 0 < 0.05, indicating that at the significant level α = 0.05, the regression of the multiple linear regression model is significant. Table 6 illustrates that the *p* values of all three factors are less than 0.05. All three factors significantly affect the plasticity zone of the roadway surrounding rock.

**Table 6:** Analysis of variance of plastic zone volume regression model.

Source	Degrees of freedom	Adj SS	Adj MS	F	P
Regress	3	687,391	229,130	58.2	0
Intensity	1	526,826	526,826	133.82	0
Frequency	1	92,208	92,208	23.42	0
Time	1	68,357	68,357	17.36	0.001
Error	12	47,243	3937		
Total	15	734,634			

Adj SS is the adjusted sum of squares; Adj MS is the squared sum of deviations; F is the test value; P is the significant difference value.

**Figure 23:**
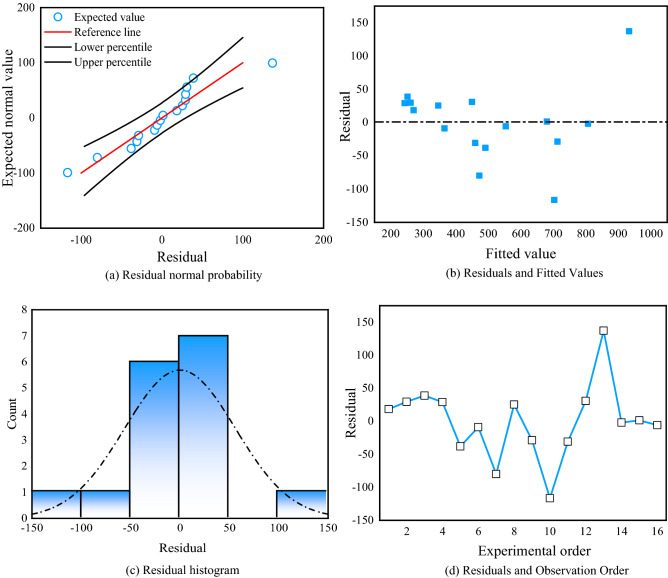
Residual plot of the plastic zone regression model.

Figure 23 shows the residuals of the regression model for the volume of the plastic zone. Figure 23a shows the residual normal probability plot, which conforms to a normal distribution and shows no outliers. Figure 23b shows a graph of residuals and observation order. The residuals fluctuate randomly, indicating that the residuals are independent of each other. Figure 23c shows the histogram of the residuals, which all conform to a normal distribution with no outliers. Figure 23d shows a graph of residuals and observation order, with random fluctuations in the residual values, indicating that the residuals are independent of each other. In summary, the regression of the plastic zone volume multiple regression model was statistically tested to be significant.

### Discussion

Deep mining of the surrounding rock is affected by high stresses and varying degrees of dynamic disturbances^49,50^. Controlling roadway surrounding rock under dynamic disturbance has become a hot research topic^51^. Since the intensity, frequency, and time of dynamic disturbance cannot be obtained under the existing technical conditions, numerical simulation software^52^ was used to study the evolution law of deformation and failure of the roadway surrounding rock under dynamic disturbance.

It was found that the greater the intensity, the lower the frequency, and the longer the duration of the disturbance, the greater the deformation and the more serious the failure of the surrounding rock. The study’s results with some scholars^53^ were mutually verified. In this paper, multiple linear regression models are used to consider various factors, and the fitting effect is more accurate compared with the literature^54^.

However, this paper does not consider the direction and location of the dynamic disturbance. Subsequent studies should be considered to carry out more numerical simulation studies to provide more reliable support for controlling deep surrounding rocks.

## Conclusions

1. In this paper, based on the three-dimensional Lagrangian finite difference method, the deformation, and failure of the roadway surrounding rock under dynamic disturbance were studied from both single-factor and multi-factor perspectives. The greater the intensity, the lower the frequency, and the longer the duration of the disturbance, the greater the deformation and the more serious the failure of the surrounding rock. Dynamic disturbance has a significant impact on the deformation of the roof, and the surrounding rock is mainly shear failure
2. Multi-factor study was realized by orthogonal experimental design, and the sensitivity of the factors influencing the deformation and failure of the roadway surrounding rock under dynamic disturbance was analyzed by applying variance and range. The degree of influence of deformation of the roof is time > intensity > frequency in order. The impact of the plastic zone volume is intensity > frequency > time in order.
3. The multiple linear regression equations of roof deformation and plastic zone volume are established respectively: y = 90.8 − 72.55x_1_ + 28.03x_2_ − 92.83x_3_, y = 90.8 − 72.55x_1_ + 28.03x_2_ − 92.83x_3_. The regression equations are statistically tested to be significant, and can effectively predict the deformation and failure of surrounding rocks under dynamic disturbance.

## Supplementary Information

Supplementary Information.

## Data Availability

The data used to support the findings of this study are included within the article.
